# Guardians of immunity: neutrophils and their role in Ebola virus infection

**DOI:** 10.1097/MS9.0000000000003966

**Published:** 2025-09-23

**Authors:** Emmanuel Ifeanyi Obeagu, Christian C. Ezeala

**Affiliations:** Department of Biomedical and Laboratory Science, Africa University, Mutare, Zimbabwe

**Keywords:** Ebola virus, hemorrhagic fever, immune response, neutrophils, viral pathogenesis

## Abstract

Ebola virus (EBOV), a member of the *Filoviridae family*, causes severe hemorrhagic fever in humans with high mortality rates. This review examines the role of neutrophils, key components of the innate immune system, in EBOV infection. Neutrophils are the first responders to infection and inflammation, performing functions such as phagocytosis, degranulation, and the formation of neutrophil extracellular traps (NETs). Despite their importance in combating bacterial and fungal infections, their role in viral infections, particularly EBOV, is complex and not fully understood. These cells contribute to both protective and pathogenic processes. On one hand, neutrophils can directly neutralize the virus and limit its spread through phagocytosis and NET formation. On the other hand, excessive neutrophil activation can exacerbate inflammation and tissue damage, contributing to the severe clinical manifestations of Ebola virus disease (EVD), such as hemorrhage and multi-organ failure. This review highlights the need for further research into the interactions between neutrophils and EBOV, which could inform the development of novel therapeutic approaches and improve clinical outcomes for patients with EVD.

## Introduction

EBOV is a highly virulent pathogen belonging to the Filoviridae family, known for causing severe outbreaks of hemorrhagic fever with high mortality rates[1]. The virus primarily affects humans and nonhuman primates, leading to Ebola virus disease (EVD), characterized by severe systemic illness, multi-organ failure, and often fatal outcomes. Since its discovery in 1976, multiple outbreaks have occurred, predominantly in West and Central Africa, with significant impacts on public health, social structures, and economies. Despite extensive research, the pathogenesis of EBOV remains only partially understood, particularly concerning the roles of various immune cells in disease progression and resolution. Neutrophils, the most abundant white blood cells in the human bloodstream, are essential components of the innate immune system[2]. They serve as the first line of defense against infections, responding rapidly to sites of infection or injury. These cells are characterized by their multilobed nuclei and granules filled with enzymes and antimicrobial proteins. Neutrophils are traditionally known for their role in combating bacterial and fungal infections through mechanisms such as phagocytosis, degranulation, and the formation of NETs. During an EBOV infection, the virus targets various cell types, including macrophages, dendritic cells, and endothelial cells, leading to widespread tissue damage and dysregulated immune responses[3]. The resulting cytokine storm and systemic inflammation are hallmarks of severe EVD, contributing to vascular leakage, hemorrhage, and multi-organ failure. While much attention has been given to the roles of macrophages and dendritic cells in EBOV pathogenesis, the contribution of neutrophils to the disease process has received comparatively less focus. Emerging evidence suggests that neutrophils play a dual role in EBOV infection, contributing to both antiviral defense and disease exacerbation[4]. On one hand, neutrophils can help control viral spread through phagocytosis of infected cells, release of antimicrobial peptides, and formation of NETs that trap and neutralize viral particles. These functions are critical for the initial containment of the infection and preventing its systemic dissemination. On the other hand, excessive neutrophil activation and degranulation can result in the release of toxic molecules that damage host tissues, exacerbate inflammation, and contribute to the severe clinical manifestations of EVD.

**HIGHLIGHTS**

- Neutrophils act as first responders in Ebola, mediating viral containment.
- Excessive NETSs and ROS drive vascular damage and coagulopathy.
- Cytokine amplification fuels immunopathology.
- Dual roles influence survival outcomes.
- Targeted modulation offers therapeutic potential.

The recruitment and activation of neutrophils in response to EBOV infection are mediated by various chemotactic signals and inflammatory mediators, such as interleukin-8 (IL-8) and leukotriene B4 (LTB4)[5]. Elevated levels of these mediators, along with increased neutrophil counts and activation markers, have been observed in the blood and tissues of EVD patients. This suggests a robust neutrophil response to EBOV infection, but the balance between their protective and pathogenic roles remains a critical area of investigation. Neutrophil extracellular traps (NETs), composed of DNA fibers and antimicrobial proteins, is another important aspect of neutrophil function. While NETs can effectively trap and neutralize pathogens, their formation can also promote thrombosis and endothelial damage, contributing to the hemorrhagic features of EVD[6]. The dual nature of NETs highlights the complexity of neutrophil involvement in EBOV infection, where beneficial antiviral effects must be weighed against potential tissue-damaging consequences. Additionally, the interaction between neutrophils and EBOV is complex, with some evidence suggesting that the virus can evade neutrophil responses or even manipulate neutrophil functions to its advantage[7]. For instance, EBOV may inhibit neutrophil activation or induce apoptosis, reducing the effectiveness of these cells in controlling the infection. Understanding these interactions is crucial for developing therapeutic strategies that can enhance the protective functions of neutrophils while mitigating their harmful effects. The clinical implications of neutrophil involvement in EBOV infection are significant (Table 1)^[8,9]^.

**Table 1:** Comparison of neutrophil responses in ebola virus disease vs. other viral infections

Feature	Ebola Virus Disease (EVD)	Influenza	SARS-CoV-2 (COVID-19)
Neutrophil count	Often elevated; high NLR linked to mortality	Elevated in severe disease	Elevated in severe COVID-19; NLR predictive of severity
NETs	Mixed role: antiviral defense vs. endothelial injury/DIC	Associated with lung tissue damage	Implicated in thrombosis and ARDS
ROS production	Excessive, contributes to vascular leakage	Moderate; contributes to lung injury	High ROS linked to cytokine storm and tissue injury
Cytokine role	Amplifies cytokine storm; IL-1β, TNF-α prominent	Drives lung inflammation	Drives cytokine storm (IL-6, IL-8, TNF-α)
Translational insight	Potential biomarker (NLR), target for modulation of NETs/ROS	Neutrophil-targeted therapies tested (e.g., antioxidants)	Trials targeting NETs, IL-8, and neutrophil trafficking ongoing

## Aim

This review aims to provide a comprehensive overview of the current understanding of neutrophils in the context of Ebola virus infection.

## Methods

### Literature search strategy

We conducted a comprehensive literature search to identify relevant publications on neutrophils and their role in EVD. The search was performed in PubMed, Scopus, and Web of Science databases, covering the period from January 2000 to June 2025. The following keywords and MeSH terms were used in various combinations: *“Ebola virus,” “Ebola virus disease,” “neutrophils,” “granulocytes,” “NETs,” “neutrophil extracellular traps,” “cytokine storm,” “innate immunity,”* and *“immune response.”* Reference lists of selected articles and relevant reviews were also screened to capture additional studies.

### Inclusion and exclusion criteria

Studies were included if they met the following criteria:

1. Reported original findings on neutrophils in the context of Ebola virus infection (human, animal, or in vitro models).
2. Addressed neutrophil biology (recruitment, NETs, cytokine production, ROS, or interactions with other immune cells).
3. Published in English in peer-reviewed journals.

We excluded:

- Editorials, commentaries, and conference abstracts without primary data.
- Studies focusing solely on other immune cells without neutrophil relevance.
- Duplicates across databases.

### Risk of bias assessment

Given the heterogeneity of study designs, we performed a qualitative risk of bias appraisal. For human studies, small sample sizes, outbreak conditions, and lack of standardized protocols were common limitations. Animal studies were constrained by species differences and biosafety restrictions. *In vitro* studies often lacked validation in physiologic systems. These limitations are acknowledged in the “Discussion” section and considered when drawing conclusions.

### Limitations

This review has several limitations that should be acknowledged. First, although we performed a structured and transparent literature search, the available evidence base on neutrophils in EVD remains limited. Most human data come from small observational cohorts conducted under outbreak conditions, where logistical and biosafety constraints restricted standardized sampling and follow-up. Consequently, conclusions regarding neutrophil dynamics in clinical EVD should be interpreted with caution. Second, a substantial proportion of the evidence derives from animal models (notably nonhuman primates and murine studies) and *in vitro* experiments, which, while valuable, may not fully replicate the complexity of human infection. Extrapolation of findings across species must therefore be approached carefully.

Third, heterogeneity in study design – including differences in experimental models, outcome measures, and laboratory techniques – limits direct comparability of results. For example, studies evaluating NETs or reactive oxygen species (ROS) production used diverse methodologies, making synthesis across reports challenging. Fourth, we did not conduct a formal systematic review or meta-analysis; instead, this is a narrative review designed to synthesize emerging insights. While this approach allows flexibility in integrating mechanistic, preclinical, and clinical perspectives, it does not provide quantitative pooled estimates of effect.

Finally, the risk of bias in the included studies was not uniformly assessed using standardized tools due to the wide range of study types (human, animal, *in vitro*). Although we qualitatively appraised methodological limitations, residual bias cannot be excluded. Despite these limitations, this review highlights critical knowledge gaps, underscores the dual protective and pathogenic roles of neutrophils in EVD, and provides a foundation for future mechanistic and translational research.

### Neutrophil biology and function

Neutrophils are a type of white blood cell and the most abundant leukocytes in the human bloodstream, playing a pivotal role in the innate immune response[10]. They are characterized by their multilobed nuclei and an arsenal of granules containing enzymes, antimicrobial peptides, and other bioactive molecules. These cells are produced in the bone marrow and have a short lifespan, typically circulating in the blood for 6-12 hours before migrating to tissues, where they can survive for an additional 1–2 days. The recruitment of neutrophils to sites of infection or injury is a highly regulated process known as chemotaxis[11]. This process is orchestrated by a variety of chemokines and signaling molecules, such as IL-8, LTB4, and complement fragments (e.g., C5a). These chemotactic signals are produced by infected or damaged tissues and attract neutrophils from the bloodstream to the site of infection. Upon sensing these signals, neutrophils undergo shape changes and directional movement, enabling them to traverse the endothelium and migrate to affected areas. One of the primary functions of neutrophils is phagocytosis, the process of engulfing and digesting pathogens and debris[12]. Neutrophils recognize pathogens through pattern recognition receptors (PRRs) that detect pathogen-associated molecular patterns (PAMPs). Upon binding to these patterns, neutrophils engulf the pathogen into a phagosome, which then fuses with granules containing antimicrobial substances. These granules release enzymes such as myeloperoxidase, lysozyme, and proteases, which help degrade and kill the ingested microbes.

In addition to phagocytosis, neutrophils can release their granule contents into the extracellular space through a process known as degranulation[13]. This mechanism allows neutrophils to combat extracellular pathogens and contribute to the inflammatory response. The granules contain a variety of antimicrobial peptides, enzymes, and other proteins that can directly kill pathogens, modulate the immune response, and interact with other immune cells. However, degranulation can also result in tissue damage if the released enzymes and ROS are not tightly regulated. A relatively recent discovery in neutrophil biology is the formation of NETs. NETs are networks of extracellular fibers composed of DNA, histones, and antimicrobial proteins. These structures can trap and kill pathogens, preventing their spread and aiding in their clearance. NET formation, or NETosis, is a distinct form of cell death that is initiated by various stimuli, including microbial components, cytokines, and immune complexes. While NETs are an important defense mechanism, their formation can also have detrimental effects, such as promoting thrombosis and contributing to tissue damage in inflammatory conditions. Neutrophils are short-lived cells, and their timely apoptosis (programmed cell death) and clearance are crucial for resolving inflammation and preventing excessive tissue damage. Apoptotic neutrophils are recognized and engulfed by macrophages in a process known as efferocytosis, which helps to limit inflammation and promote tissue repair. Dysregulation of neutrophil apoptosis can lead to prolonged inflammation and has been implicated in various chronic inflammatory diseases[13].

While neutrophils are well-studied in the context of bacterial and fungal infections, their role in viral infections is less clear and more complex. Neutrophils can respond to viral infections through direct antiviral activities, such as phagocytosing virus-infected cells, producing antiviral cytokines, and forming NETs that can trap viral particles. However, their role can also be double-edged, as excessive neutrophil activation and degranulation can contribute to immunopathology and tissue damage, exacerbating disease severity[10]. Neutrophils do not act in isolation but interact with various other immune cells, including macrophages, dendritic cells, T cells, and B cells. These interactions can influence the overall immune response, with neutrophils contributing to the activation, recruitment, and regulation of other immune cell types. For example, neutrophils can produce cytokines and chemokines that recruit and activate macrophages and lymphocytes, shaping the adaptive immune response[11]. The functions of neutrophils are tightly regulated by various signaling pathways and feedback mechanisms to ensure an effective yet controlled immune response. Dysregulation of neutrophil activity, whether through excessive activation or impaired apoptosis, can lead to pathological conditions[12]. Neutrophils are central players in the innate immune response, and their functions have significant clinical implications. Their role in various infectious and inflammatory diseases makes them potential targets for therapeutic intervention. Modulating neutrophil recruitment, activation, or apoptosis could provide therapeutic benefits in conditions ranging from acute infections to chronic inflammatory diseases and autoimmune disorders. However, therapeutic strategies must carefully balance enhancing protective functions while minimizing potential tissue-damaging effects[13].

### Ebola virus pathogenesis

EBOV, a member of the Filoviridae family, is a highly virulent pathogen responsible for severe hemorrhagic fever outbreaks in humans and non-human primates[14]. The pathogenesis of EVD involves a complex interplay between viral replication, immune response dysregulation, and widespread tissue damage. The pathogenesis of EBOV begins with the virus entering the host through mucosal surfaces or breaches in the skin[15]. The primary targets for EBOV are macrophages and dendritic cells, which are among the first cells to encounter the virus. The glycoprotein (GP) on the surface of EBOV facilitates viral entry by binding to cellular receptors such as Niemann-Pick C1 (NPC1) and triggering endocytosis. Once inside the host cell, the virus escapes the endosome and releases its RNA genome into the cytoplasm, initiating replication and transcription. Following initial replication at the site of entry, EBOV spreads to regional lymph nodes and subsequently to the bloodstream, leading to systemic viremia. The virus disseminates widely throughout the body, infecting a variety of cell types, including monocytes, macrophages, dendritic cells, hepatocytes, endothelial cells, and fibroblasts. This broad tissue tropism contributes to the widespread tissue damage and multi-organ dysfunction observed in EVD. One of the hallmarks of EBOV pathogenesis is the profound dysregulation of the host immune response[16]. EBOV infection triggers a strong pro-inflammatory response characterized by the release of cytokines and chemokines, commonly referred to as a “cytokine storm.” Key cytokines involved include tumor necrosis factor-alpha (TNF-α), interleukin-6 (IL-6), and interleukin-1 beta (IL-1β). This excessive inflammatory response leads to widespread endothelial activation and dysfunction, contributing to vascular leakage and hemorrhage.

EBOV has evolved several strategies to evade the host immune response, allowing it to replicate and spread unchecked. The viral proteins VP24 and VP35 play critical roles in this immune evasion[17]. VP35 inhibits the induction of type I interferon (IFN) responses by targeting the RIG-I-like receptor (RLR) signaling pathway, while VP24 interferes with the JAK-STAT signaling pathway, preventing the activation of antiviral genes. These mechanisms enable the virus to avoid detection and clearance by the host immune system, facilitating sustained viral replication. The severe hemorrhagic manifestations of EVD are a result of the combined effects of viral replication, immune dysregulation, and direct endothelial cell damage[18]. The EBOV GP is cytotoxic to endothelial cells, inducing cell detachment, apoptosis, and barrier dysfunction. Additionally, the overactivation of neutrophils and the formation of NETs contribute to endothelial damage and thrombosis. The result is a disruption of the vascular integrity, leading to hemorrhage and disseminated intravascular coagulation (DIC). The widespread tissue damage and systemic inflammatory response in EVD lead to multi-organ failure, which is a major cause of mortality in infected individuals[19]. The liver, kidneys, and adrenal glands are particularly affected. Hepatocellular necrosis impairs the synthesis of clotting factors, exacerbating hemorrhagic tendencies. Renal dysfunction results from acute tubular necrosis and impaired blood flow, leading to acute kidney injury. Adrenal insufficiency, caused by direct viral invasion and necrosis, contributes to hypotension and shock.

**Figure 1.**
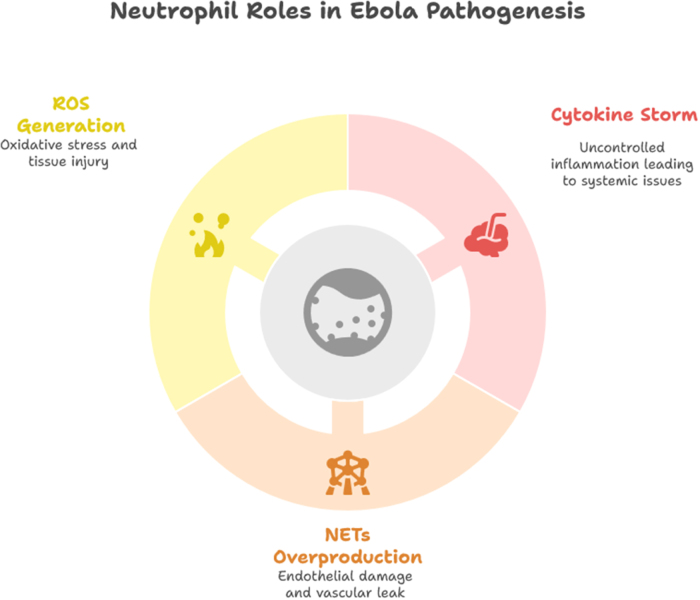
Neutrophil roles in Ebola pathogenesis.

While the innate immune response is crucial for initial control of EBOV infection, the adaptive immune response plays a vital role in clearing the virus and providing long-term immunity. The humoral immune response, mediated by antibodies, targets the viral glycoprotein and neutralizes the virus. The cellular immune response, involving CD8 + cytotoxic T lymphocytes (CTLs), is essential for eliminating infected cells. However, the early and overwhelming nature of EBOV infection can impair the development of an effective adaptive immune response, contributing to the high mortality rate[15]. Current treatment approaches include supportive care, such as fluid resuscitation and electrolyte management, and antiviral therapies like remdesivir. In addition, monoclonal antibodies targeting the viral glycoprotein have shown promise in neutralizing the virus and improving survival. Therapeutic interventions aimed at modulating the immune response, such as corticosteroids or cytokine inhibitors, may help mitigate the excessive inflammation and improve clinical outcomes. Vaccination is a key strategy for preventing EBOV outbreaks and controlling the spread of the virus[20]. The rVSV-ZEBOV vaccine, based on a recombinant vesicular stomatitis virus expressing the EBOV glycoprotein, has shown high efficacy in preventing EVD. Mass vaccination campaigns and ring vaccination strategies have been implemented successfully in outbreak settings. Continued research and development of vaccines, as well as strategies to enhance vaccine delivery and distribution, are essential for effective EBOV control (Fig. 1).

### Neutrophils in Ebola virus infection

#### Recruitment and activation

The recruitment and activation of neutrophils are crucial processes in the host defense against infections, including those caused by EBOV[21]. Neutrophils are the first responders to sites of infection and inflammation, and their timely recruitment and activation are essential for initiating the immune response. The recruitment of neutrophils to sites of infection is primarily driven by chemotactic signals, which are molecules that create a chemical gradient guiding neutrophils from the bloodstream to the site of infection. During EBOV infection, infected cells and immune cells release a variety of chemokines and other signaling molecules that attract neutrophils. Key chemotactic signals involved in neutrophil recruitment include:

- Interleukin-8 (IL-8) is a potent chemokine produced by various cell types, including macrophages, endothelial cells, and fibroblasts, in response to infection and inflammation. IL-8 binds to its receptors, CXCR1 and CXCR2, on neutrophils, inducing chemotaxis and guiding them to the site of infection.

-Leukotriene B4 (LTB4) is a lipid mediator derived from arachidonic acid and produced by activated leukocytes. It binds to its receptor, BLT1, on neutrophils, promoting their migration towards higher concentrations of LTB4 at the infection site.

-Complement Fragments (C5a): activated during infection, leading to the generation of complement fragments such as C5a. C5a is a powerful chemoattractant for neutrophils, binding to the C5a receptor (C5aR) on their surface and directing their movement.

These chemotactic signals create a gradient that neutrophils follow, allowing them to exit the bloodstream, traverse the endothelial barrier, and migrate through the extracellular matrix to reach the site of EBOV infection[22]. Upon arrival, neutrophils undergo activation, a process that prepares them to perform their antimicrobial functions. Neutrophil activation involves a series of signaling events triggered by the binding of various receptors to their ligands. Neutrophils express a variety of PRRs, such as Toll-like receptors (TLRs) and NOD-like receptors (NLRs), which recognize pathogen-associated molecular patterns (PAMPs) present on EBOV[23]. Binding of these receptors to their ligands activates signaling pathways that lead to neutrophil activation. Neutrophils also express Fc receptors that bind to the Fc region of antibodies. During EBOV infection, antibodies produced by the adaptive immune system can opsonize the virus, marking it for destruction[24]. Binding of these antibody–virus complexes to Fc receptors on neutrophils triggers phagocytosis and activation. In addition to C5a, other complement fragments such as C3b can opsonize EBOV particles. Neutrophils express complement receptors (e.g., CR1, CR3) that bind to these opsonized particles, leading to phagocytosis and activation.

Activated neutrophils engulf EBOV-infected cells and virus particles through phagocytosis[25]. This process involves the internalization of the pathogen into a phagosome, which then fuses with lysosomes to form a phagolysosome, where the pathogen is degraded by antimicrobial enzymes. Neutrophils contain granules filled with antimicrobial proteins and enzymes, such as myeloperoxidase, defensins, and proteases. Upon activation, neutrophils release the contents of these granules into the extracellular space through degranulation, helping to kill extracellular pathogens and modulate the immune response.

Activated neutrophils can release NETs, web-like structures composed of DNA, histones, and antimicrobial proteins. NETs Trapstrap and neutralize EBOV particles, preventing their spread and aiding in their clearance by other immune cells[26]. The activity of neutrophils is tightly regulated to ensure an effective yet controlled immune response.

Dysregulation of neutrophil recruitment and activation can lead to excessive inflammation and tissue damage. Anti-inflammatory cytokines, such as interleukin-10 (IL-10) and transforming growth factor-beta (TGF-β), help regulate neutrophil activity and prevent excessive inflammation. Neutrophils undergo programmed cell death (apoptosis) after fulfilling their functions. Apoptotic neutrophils are recognized and engulfed by macrophages in a process called efferocytosis, which helps resolve inflammation and promote tissue repair. Neutrophil activation is modulated by feedback mechanisms involving various receptors and signaling pathways, ensuring that neutrophil responses are proportional to the level of infection and inflammation. The recruitment and activation of neutrophils play crucial roles in the immune response to EBOV infection[27]. While neutrophils contribute to controlling the infection through their antimicrobial functions, excessive activation and degranulation can exacerbate inflammation and tissue damage, contributing to the severe clinical manifestations of EVD.

#### Antiviral defense mechanisms

Neutrophils, key players in the innate immune system, employ several antiviral defense mechanisms to combat infections, including those caused by the EBOV[28]. These mechanisms are critical in the initial control and eventual clearance of the virus. Phagocytosis is one of the primary defense mechanisms of neutrophils involving the engulfment and internalization of pathogens, including viruses and virus-infected cells. During EBOV infection, neutrophils recognize and bind to the virus or infected cells through PRRs that detect PAMPs. Once bound, the pathogen is engulfed into a phagosome, which then fuses with lysosomes to form a phagolysosome. Inside the phagolysosome, the pathogen is exposed to a variety of antimicrobial enzymes and ROS, leading to its degradation and destruction.

Neutrophils contain granules packed with antimicrobial proteins and enzymes, which can be released into the extracellular space through a process known as degranulation. This mechanism is especially important for combating extracellular pathogens. During EBOV infection, degranulation releases enzymes such as myeloperoxidase, defensins, and proteases, which can directly kill viral particles and infected cells[29]. Additionally, the release of these antimicrobial substances can enhance the overall immune response by recruiting and activating other immune cells.

NETs are web-like structures composed of DNA, histones, and antimicrobial proteins that neutrophils release in response to infection. NETs trap and neutralize EBOV particles, preventing their spread and facilitating their clearance by other immune cells. The formation of NETs, or NETosis, is a distinct form of cell death initiated by various stimuli, including microbial components, cytokines, and immune complexes. While NETs are effective in trapping and killing pathogens, their formation can also have detrimental effects, such as promoting thrombosis and contributing to tissue damage.

Neutrophils generate ROS as part of their antimicrobial arsenal. The production of ROS occurs through the activation of the NADPH oxidase complex, which generates superoxide anions that are subsequently converted into other ROS, such as hydrogen peroxide and hydroxyl radicals. These ROS can directly damage viral particles and infected cells, contributing to the control of EBOV infection. However, excessive ROS production can also lead to oxidative stress and tissue damage, highlighting the need for balanced ROS generation. In addition to their direct antiviral activities, neutrophils produce a variety of cytokines and chemokines that modulate the immune response.

During EBOV infection, neutrophils secrete pro-inflammatory cytokines, such as tumor necrosis factor-alpha (TNF-α), interleukin-1 beta (IL-1β), and interleukin-6 (IL-6). These cytokines help recruit and activate other immune cells, such as macrophages, dendritic cells, and lymphocytes, enhancing the antiviral response. However, the excessive production of pro-inflammatory cytokines can contribute to the cytokine storm observed in severe EVD cases, leading to systemic inflammation and tissue damage[29].Neutrophils alsoproduce and release a range of antiviral peptides and proteins that contribute to the control of EBOV infection. These include defensins, cathelicidins, and lactoferrin. Defensins are small cationic peptides that can disrupt viral envelopes and inhibit viral replication. Cathelicidins, such as LL-37, have broad-spectrum antiviral activity and can modulate the immune response. Lactoferrin, an iron-binding glycoprotein, has antiviral properties and can inhibit viral entry and replication. The combined action of these peptides and proteins enhances the antiviral defense mechanisms of neutrophils.

Neutrophils do not act in isolation; they interact with various other immune cells to coordinate an effective antiviral response. Neutrophils can influence the function of macrophages, dendritic cells, T cells, and B cells through the release of cytokines, chemokines, and other signaling molecules. For example, neutrophil-derived cytokines can enhance the antigen-presenting capabilities of dendritic cells, promoting the activation of T cells and the subsequent adaptive immune response. Additionally, neutrophils can provide help to B cells, facilitating the production of antiviral antibodies. Neutrophils can participate in antibody-dependent cellular cytotoxicity (ADCC), a mechanism by which they recognize and kill virus-infected cells that are coated with antibodies. During EBOV infection, antibodies produced by the adaptive immune system can opsonize infected cells[30]. Neutrophils express Fc receptors that bind to the Fc region of these antibodies, triggering the release of cytotoxic granules and the destruction of the infected cells. ADCC enhances the antiviral activity of neutrophils and contributes to the clearance of EBOV-infected cells. While neutrophils are crucial for initiating and sustaining the antiviral response, their activity must be tightly regulated to prevent excessive inflammation and tissue damage. Anti-inflammatory cytokines, such as interleukin-10 (IL-10) and transforming growth factor-beta (TGF-β), help modulate neutrophil activity and limit excessive inflammation. Additionally, the resolution of neutrophil-mediated inflammation involves the induction of apoptosis (programmed cell death) in neutrophils and their subsequent clearance by macrophages through efferocytosis. This process helps resolve inflammation and promote tissue repair.

The antiviral defense mechanisms of neutrophils are critical role in controlling EBOV infection and influencing disease outcomes[31]. Therapeutic strategies that enhance the antiviral functions of neutrophils, while minimizing their potential for causing tissue damage, could improve patient outcomes. For example, interventions that promote NET formation or boost the production of antiviral peptides could enhance viral clearance. Conversely, strategies that mitigate excessive neutrophil activation and degranulation could help reduce inflammation and tissue damage.

#### Contribution to pathogenesis

While neutrophils play a vital role in the initial immune response to EBOV infection, their involvement in the pathogenesis of EVD can be both protective and detrimental[32]. This duality is a significant aspect of EVD’s complexity. Neutrophils contribute to controlling the virus and shaping the immune response, but their overactivation and dysregulation can exacerbate disease severity, leading to tissue damage and multi-organ failure. During EBOV infection, the rapid and massive recruitment of neutrophils to sites of infection can lead to an excessive inflammatory response, commonly referred to as a cytokine storm[33]. This uncontrolled release of pro-inflammatory cytokines, such as tumor necrosis factor-alpha (TNF-α), interleukin-1 beta (IL-1β), and interleukin-6 (IL-6), results in widespread endothelial activation and dysfunction. The excessive inflammation can cause increased vascular permeability, leading to edema, hemorrhage, and multi-organ dysfunction. The cytokine storm is a hallmark of severe EVD and is closely associated with poor clinical outcomes. NETs are critical for trapping and neutralizing EBOV particles[34]. However, the formation of NETs can also have deleterious effects. NETs, composed of DNA, histones, and antimicrobial proteins, can promote thrombosis and contribute to tissue damage if not properly regulated. The release of NETs during EBOV infection can exacerbate vascular damage and inflammation, further compromising the integrity of blood vessels and contributing to the severe hemorrhagic manifestations of EVD.

Neutrophils produce ROS as part of their antimicrobial defense. While ROS can help control viral replication, excessive ROS production can lead to oxidative stress and damage to host tissues. During EBOV infection, the overproduction of ROS by activated neutrophils can result in significant tissue damage, particularly in the liver, kidneys, and vascular endothelium. This oxidative damage contributes to the pathogenesis of EVD and the development of multi-organ failure.

The degranulation of neutrophils releases a variety of proteolytic enzymes, such as elastase and myeloperoxidase, which are essential for killing pathogens. However, these enzymes can also degrade extracellular matrix components and damage host tissues. In the context of EBOV infection, neutrophil degranulation can lead to the destruction of endothelial cells, disruption of the vascular barrier, and promotion of hemorrhage. This tissue damage is a significant factor in the severe clinical manifestations of EVD, including bleeding and organ dysfunction[18]. Neutrophils can contribute to immunopathology by perpetuating a cycle of inflammation and tissue damage. The release of pro-inflammatory cytokines and chemokines by neutrophils can recruit and activate additional immune cells, leading to a sustained and amplified inflammatory response. This persistent inflammation can result in chronic tissue damage and fibrosis, impairing the function of affected organs. In severe cases of EVD, the immunopathology driven by neutrophils and other immune cells can lead to systemic inflammation, septic shock, and death[19]. Neutrophils interact closely with endothelial cells, which line the blood vessels and play a crucial role in maintaining vascular integrity. During EBOV infection, the interaction between activated neutrophils and endothelial cells can lead to endothelial activation and damage. Neutrophils can release inflammatory mediators that increase endothelial permeability, promoting fluid leakage and contributing to edema and hemorrhage. Additionally, the cytotoxic effects of neutrophil-derived enzymes and ROS can directly damage endothelial cells, exacerbating vascular dysfunction.

The timely apoptosis (programmed cell death) and clearance of neutrophils are essential for resolving inflammation and promoting tissue repair. However, during EBOV infection, the dysregulation of neutrophil apoptosis can lead to prolonged inflammation and tissue damage. Delayed clearance of apoptotic neutrophils can result in secondary necrosis, further releasing pro-inflammatory mediators and perpetuating the inflammatory response. This delayed resolution of inflammation can contribute to the chronic and severe manifestations of EVD.

Neutrophils play a role in the coagulation cascade, and their dysregulation during EBOV infection can contribute to coagulopathy. The release of tissue factor and other pro-coagulant molecules by activated neutrophils can promote disseminated intravascular coagulation (DIC), a condition characterized by widespread clotting and bleeding. DIC is a common complication of severe EVD and is associated with high mortality. The interplay between neutrophil activation, NET formation, and coagulopathy underscores the complex contribution of neutrophils to the pathogenesis of EVD[15]. Interventions that reduce excessive neutrophil recruitment and activation may help mitigate the inflammatory response and tissue damage. Targeting the pathways involved in NET formation could prevent NET-mediated tissue damage and thrombosis. Antioxidant treatments may help reduce oxidative stress and tissue damage caused by excessive ROS production. Modulating the production of pro-inflammatory cytokines by neutrophils could help control the cytokine storm and reduce systemic inflammation. Promoting the timely apoptosis and clearance of neutrophils may aid in resolving inflammation and preventing chronic tissue damage[16].

Neutrophils, as first responders in the immune system, interact with the EBOV in various complex ways. These interactions can significantly influence the outcome of EVD, contributing to both the control of the virus and the pathogenesis of the disease. Neutrophils express various PRRs that recognize PAMPs present on EBOV[35]. Key PRRs involved in EBOV recognition include lTLRs, particularly TLR4 and TLR7/8, which can detect viral components such as glycoproteins and RNA. The engagement of these receptors triggers intracellular signaling pathways leading to the activation of neutrophils and the production of pro-inflammatory cytokines and chemokines. Once activated, neutrophils can phagocytose EBOV particles[36]. Phagocytosis involves the engulfment of the virus into a phagosome, which subsequently fuses with lysosomes to form a phagolysosome. The acidic and enzymatic environment within the phagolysosome facilitates the degradation of EBOV particles. However, the efficiency of this process in fully neutralizing EBOV remains a subject of ongoing research, as some studies suggest that EBOV can evade complete destruction within phagocytes. Neutrophils can release NETs in response to EBOV infection[37]. NETs are networks of extracellular fibers composed of DNA, histones, and antimicrobial proteins. They can trap and neutralize viral particles, preventing the spread of the virus. However, the formation of NETs, while beneficial in trapping EBOV, can also contribute to tissue damage and inflammation, complicating the pathogenesis of EVD.

Neutrophils produce a variety of pro-inflammatory cytokines in response to EBOV infection, including TNF-α, IL-1β, and IL-6[37]. These cytokines play a critical role in amplifying the immune response by recruiting and activating other immune cells, such as macrophages and lymphocytes. However, the excessive production of these cytokines can lead to a cytokine storm, characterized by systemic inflammation and multi-organ dysfunction, which is a major contributor to the severity of EVD. Neutrophils interact with other immune cells, such as macrophages and dendritic cells, to coordinate the immune response against EBOV. Through the release of cytokines and chemokines, neutrophils can enhance the antigen-presenting capabilities of dendritic cells, promoting the activation of T cells. Additionally, neutrophils can influence the polarization of macrophages towards a pro-inflammatory phenotype, further amplifying the immune response. Neutrophils release proteolytic enzymes and ROS upon activation, which can directly kill EBOV-infected cells. However, these antimicrobial mechanisms can also damage surrounding tissues. The release of enzymes such as elastase and myeloperoxidase, along with the production of ROS, can degrade extracellular matrix components and damage endothelial cells, contributing to the vascular leakage and hemorrhage observed in EVD. The apoptosis (programmed cell death) and subsequent clearance of neutrophils are essential for resolving inflammation and preventing chronic tissue damage. During EBOV infection, the timely induction of neutrophil apoptosis and their clearance by macrophages through efferocytosis help to limit excessive inflammation and promote tissue repair. However, dysregulation of these processes can lead to prolonged inflammation and secondary tissue damage[37]. Targeting pathways that modulate neutrophil activation and cytokine production could help reduce excessive inflammation and tissue damage. Strategies to enhance the beneficial effects of NETs while minimizing their contribution to tissue damage may improve outcomes in EVD. Antioxidant treatments could help mitigate the oxidative stress and tissue damage caused by excessive ROS production. Therapies that promote the timely apoptosis and clearance of neutrophils may aid in resolving inflammation and preventing chronic tissue damage.

#### Therapeutic implications

Neutrophils are central to the immune response against EBOV, but their overactivation and dysregulation can contribute to severe disease outcomes. Targeted therapies aimed at modulating neutrophil function and mitigating their pathological effects could enhance patient outcomes[18]. Drugs that inhibit pro-inflammatory cytokines, such as TNF-α, IL-1β, and IL-6, can help control the inflammatory response. For example, monoclonal antibodies targeting IL-6 (e.g., tocilizumab) have shown promise in other inflammatory conditions and may be beneficial in reducing systemic inflammation in EVD[19]. Agents like corticosteroids can help reduce the overall inflammatory response. However, their use must be carefully balanced to avoid suppressing the beneficial aspects of the immune response. Targeting TLRs involved in recognizing EBOV can prevent overactivation of neutrophils. Specific TLR antagonists or modulators could help in controlling the initial immune response[19]. Since NADPH oxidase is involved in ROS production, inhibitors of this enzyme could reduce oxidative stress and tissue damage while preserving the neutrophil’s ability to combat the virus. NETs are crucial for trapping and neutralizing EBOV but can also contribute to tissue damage. Developing agents that modulate NET formation, such as inhibitors of excessive NETosis, can help balance the protective and harmful effects of NETs. Enzymes or compounds that specifically degrade NETs without affecting other aspects of neutrophil function might mitigate NET-mediated damage while preserving their antiviral activity[21].

Use of anticoagulants to prevent NET-induced coagulation could help manage coagulopathy and reduce the risk of disseminated intravascular coagulation (DIC) in severe EVD cases. Excessive production of ROS by neutrophils can lead to tissue damage. Administering antioxidants, such as *N*-acetylcysteine or superoxide dismutase mimetics, can help neutralize excess ROS and reduce oxidative stress. Targeting the NADPH oxidase complex to regulate ROS production may help control oxidative damage while preserving the antimicrobial functions of neutrophils. Compounds that promote programmed cell death in neutrophils could help resolve inflammation and prevent chronic tissue damage. These agents would need to be carefully balanced to avoid excessive apoptosis. Promoting the clearance of apoptotic neutrophils by macrophages through agents that enhance efferocytosis could aid in resolving inflammation and facilitating tissue repair. Using anticoagulants to manage DIC and other coagulopathic complications associated with severe EVD[38]. Developing inhibitors that specifically target the pro-coagulant factors released by neutrophils could help mitigate coagulopathy without broadly suppressing coagulation. Integrating anti-inflammatory agents with antioxidants to simultaneously address excessive inflammation and oxidative stress. Employing therapies that simultaneously target PRRs, NETs, ROS, and coagulopathy to address multiple aspects of neutrophil-mediated pathogenesis.

#### Neutrophil-targeted insights: translating immunology into therapeutic strategies and public health outcomes

Understanding the dualistic roles of neutrophils in Ebola virus infection provides a crucial foundation for therapeutic innovation and improved clinical management. Neutrophils are both indispensable first responders and potential drivers of pathology, and this paradox positions them as attractive, yet challenging, targets for intervention. From a therapeutic perspective, modulating neutrophil responses could mitigate the immunopathology that contributes to EVD mortality. Strategies aimed at tuning, rather than suppressing, neutrophil activity may offer the greatest benefit. For example, selective inhibition of NET overproduction or excessive ROS generation could reduce vascular injury and disseminated intravascular coagulation without undermining early antiviral functions. Similarly, targeted cytokine modulation – such as blocking IL-8 or GM-CSF to prevent neutrophil hyperrecruitment – might attenuate the cytokine storm while preserving baseline immune defense. Emerging biologics, including monoclonal antibodies and small-molecule inhibitors of neutrophil activation pathways, represent promising avenues for translational research^[39,40]^.

At the clinical level, neutrophil-derived markers may serve as prognostic tools for triaging patients and guiding therapy. Elevated neutrophil-to-lymphocyte ratio (NLR), excessive circulating NET fragments, or aberrant degranulation markers could help identify patients at risk of severe immunopathology, thereby prioritizing them for intensive monitoring and adjunctive therapies. Integrating such biomarkers into clinical protocols could shift the paradigm from reactive to anticipatory care, improving survival outcomes in resource-limited settings^[41,42].^ The implications also extend to public health and outbreak response. In regions where Ebola emerges, understanding neutrophil dysregulation can inform supportive care guidelines, especially in the absence of universal access to antivirals or vaccines. For example, therapies that stabilize vascular integrity or limit coagulopathy – both indirectly tied to neutrophil-driven injury – could reduce case fatality rates when deployed as part of outbreak treatment packages. Furthermore, neutrophil biomarkers may enhance epidemiological surveillance, offering a simple, blood-based means to stratify disease severity and allocate scarce medical resources during epidemics^[43,44].^ Bridging immunology with public health, neutrophil research underscores the importance of precision immunomodulation in Ebola management. Rather than broadly dampening the immune system, the next generation of interventions should aim to recalibrate neutrophil activity – amplifying their protective roles in viral clearance while minimizing their contribution to vascular leak, systemic inflammation, and organ failure. Such a nuanced approach not only refines therapeutic strategies but also strengthens preparedness for future outbreaks, where rapid clinical decision-making and targeted interventions are essential^[45,46]^.

#### Contrasting protective and pathogenic roles of neutrophils in Ebola virus infection

Neutrophils are among the earliest immune cells recruited to sites of EBOV infection, acting as both guardians and, paradoxically, contributors to disease progression. Their functions span a wide spectrum, with protective mechanisms crucial for viral containment on one end and pathogenic consequences that fuel immunopathology on the other. On the protective side, neutrophils serve as the first line of defense by rapidly migrating to infected tissues and deploying mechanisms such as phagocytosis, degranulation, and the release of antimicrobial peptides. These responses help limit initial viral replication and spread. In addition, neutrophils generate pro-inflammatory cytokines including TNF-α and IL-1β, which act as important signals to recruit and activate other immune cells. Controlled production of ROS further contributes to pathogen clearance, while the formation of NETs can immobilize viral particles and reduce systemic dissemination. Through cross-talk with dendritic cells and T lymphocytes, neutrophils can also bridge innate and adaptive immunity, ultimately contributing to viral clearance and improved survival in some hosts (Table 2)[45].

**Table 2:** Dual roles of neutrophils in Ebola virus disease

Neutrophil function	Protective role	Pathogenic role	Evidence source
Phagocytosis	Engulfment of opsonized viral particles, limiting spread	Inefficient against non-opsonized EBOV	Animal and *in vitro* studies
Degranulation	Release of antimicrobial peptides	Excess protease release damages endothelium, contributing to vascular leakage	*In vitro* + animal
Reactive oxygen species (ROS)	Oxidative killing of viral components	Excess ROS amplifies cytokine storm and tissue injury	*In vitro* + human samples
Neutrophil extracellular traps (NETs)	Trap viral particles, restrict dissemination	Promote thrombosis, endothelial dysfunction, and DIC	Murine + NHP models
Cytokine secretion	Recruit and activate other immune cells	Amplify cytokine storm, worsening immunopathology	Human observational + in vitro
Crosstalk with lymphocytes	Support adaptive immunity in early infection	Contribute to T-cell apoptosis and immune paralysis	Human + animal

**Figure 2.**
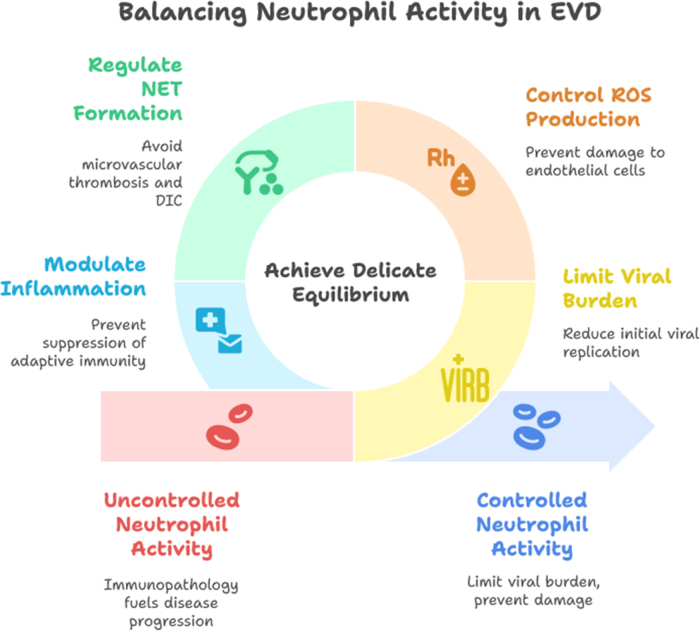
Contrasting protective and pathogenic roles of neutrophils in Ebola virus infection.

However, these same effector functions, when dysregulated, can drive pathological outcomes characteristic of severe EVD. Neutrophil hyperactivation contributes to the massive cytokine release that underpins the “cytokine storm,” a hallmark of fatal cases. Excessive ROS production, while antimicrobial, also damages host endothelial cells, aggravating vascular permeability and contributing to hemorrhagic manifestations. Similarly, uncontrolled NET formation, rather than being protective, can promote microvascular thrombosis and DIC, exacerbating organ failure. Infiltration of large numbers of neutrophils into vital organs such as the liver, lungs, and vascular endothelium further amplifies tissue destruction and impairs organ function. Moreover, sustained neutrophil-driven inflammation can suppress adaptive immune responses by impairing dendritic cell function and antigen presentation, thereby weakening the host’s ability to mount long-lasting immunity[46]. The dual nature of neutrophil responses highlights their role as a double-edged sword in EVD. While their rapid deployment is indispensable for early containment of viral replication, excessive or uncontrolled activity often tips the balance toward immunopathology. Survival outcomes may therefore depend on the host’s ability to achieve a delicate equilibrium – where neutrophil activity is sufficient to limit viral burden without precipitating the collateral damage that fuels hemorrhage, shock, and multi-organ failure (Fig. 2).

#### Challenges and future directions

Despite significant progress in understanding the immunopathology of EVD, the role of neutrophils remains underexplored and fraught with conceptual and technical challenges. A major limitation lies in the difficulty of studying neutrophils during acute EVD outbreaks, where patient access is restricted and biosafety constraints limit the scope of mechanistic investigations. Most available data are extrapolated from animal models or in vitro studies, which, while valuable, may not fully recapitulate the dynamics of human disease. Additionally, the short lifespan and heterogeneity of neutrophil subsets complicate efforts to dissect their precise contributions to Ebola pathogenesis. For instance, distinguishing between protective neutrophil functions – such as early viral containment – and pathogenic consequences—such as excessive NET release or oxidative injury—remains a formidable task[44]. Another challenge stems from the overlap between neutrophil responses and the broader cytokine storm characteristic of EVD. Because multiple immune cell types produce overlapping mediators, it is difficult to assign causality to neutrophils in driving endothelial leak, coagulopathy, or systemic inflammation. This lack of clarity hampers the translation of basic immunological insights into targeted interventions. Furthermore, therapeutic strategies aimed at modulating neutrophil activity face a delicate balance: suppressing harmful effects such as uncontrolled NETosis or ROS production without undermining their frontline antiviral defense[45].

Several avenues for research hold promise. High-resolution immunoprofiling, including single-cell transcriptomics and proteomics, could unravel the heterogeneity of neutrophil subsets during EVD and clarify their context-dependent roles. Longitudinal sampling of survivors and patients with varying disease severities may reveal signatures of neutrophil-driven protection versus pathology, offering prognostic biomarkers for clinical management. Moreover, integrating neutrophil biology into the development of host-directed therapies – such as inhibitors of NET formation, ROS scavengers, or cytokine-targeting biologics – could help mitigate immune-mediated damage without impairing viral clearance[46]. From a public health perspective, future directions must also address how insights into neutrophil biology can be operationalized during outbreaks. Rapid, field-deployable assays to assess neutrophil activation or NET markers could improve triage and risk stratification in resource-limited settings. Additionally, clinical trials evaluating adjunctive immunomodulatory therapies should explicitly consider neutrophil-related endpoints to avoid unintended consequences on innate immunity[47].

## Conclusion

Neutrophils are crucial components of the innate immune system, playing a dual role in the pathogenesis of EVD. Their primary functions – phagocytosis, degranulation, ROS production, and extracellular trap formation – are essential for controlling EBOV infection. However, the dysregulation of these processes can contribute significantly to the severity of EVD. Understanding these interactions provides valuable insights into potential therapeutic strategies. The complexity of neutrophil responses in EVD highlights the need for targeted interventions. Excessive activation of neutrophils can lead to a cytokine storm, increased oxidative stress, and tissue damage, exacerbating the clinical manifestations of the disease. Therefore, therapeutic strategies must aim to balance the protective functions of neutrophils while mitigating their pathogenic effects. Approaches such as modulating inflammatory responses, regulating NET formation, controlling oxidative stress, and managing coagulopathy are essential for addressing the multifaceted role of neutrophils in EVD. Advancements in understanding neutrophil biology and their interactions with EBOV are critical for developing effective treatments. By leveraging this knowledge, it is possible to design therapies that enhance the immune response against EBOV while minimizing collateral damage. Future research should focus on optimizing these therapeutic strategies, ensuring their safety and efficacy, and exploring novel interventions that can improve patient outcomes.

## Data Availability

Not applicable as this a narrative review.

## Research registration unique identifying number (UIN)

Not applicable as this a narrative review.
